# Supplementary material for: The Efficacy of Stem Cells in Wound Healing: A Systematic Review
Source: Int J Mol Sci. 2024 Mar 5;25(5):3006. doi: 10.3390/ijms25053006 (PMC10931571; doi:10.3390/ijms25053006)
Supplement: Supplementary file 1 [file ijms-25-03006-s001.zip › Supplementary S1.pdf]

Complete Search Strings

Source	String	Date Run	References Retrieved
PubMed	("Wound Healing"[Mesh] OR "Wound healing"[tw] OR "platelet derived wound healing"[tw] OR "wound regeneration"[tw]) AND ("Stem Cells"[Mesh] OR "Adult Stem Cells"[Mesh] OR "Embryonic Stem Cells"[Mesh] OR "Stem Cell Transplantation"[Mesh] OR "Adipose Tissue/cytology"[Mesh] OR "Mesenchymal Stem Cells"[Mesh] OR "Pluripotent Stem Cells"[Mesh] OR "Mesenchymal Stem Cell Transplantation"[Mesh] OR "stem cell"[tw] OR "stem cells"[tw] OR "Progenitor Cells"[tw] OR "mother cells"[tw] OR "Colony forming units"[tw] OR "adult stem cells"[tw] OR "Adipose tissue derived stem cells"[tw] OR "bone marrow derived mesenchymal stem cells"[tw] OR "inducible pluripotent stem cells"[tw] OR "endothelial progenitor stem cells"[tw] OR "epidermal stem cells"[tw] OR "embryonic stem cells"[tw] OR "peripheral blood derived mesenchymal stem cells"[tw] OR "human umbilical cord mesenchymal stem cells"[tw] OR "skin derived stem cells"[tw] OR "Wharton's jelly mesenchymal stem cells"[tw] OR "mesenchymal stem cells"[tw] OR "Wharton's Jelly Cells"[tw] OR "Mesenchymal stromal cells"[tw] OR MSC[tw] OR ADCS[tw]) AND ("Humans"[Mesh] OR Human OR "homo sapiens"[tw]) NOT ("Animals"[Mesh] NOT ("Animals"[Mesh] AND "Humans"[Mesh]))	12/18/2023	6732
Embase	('human'/exp OR 'homo sapiens' OR 'human' OR 'human being' OR 'human body' OR 'humans' OR 'man (homo sapiens)') AND ('adult stem cell'/exp OR 'adult germline stem cells' OR 'adult stem cell' OR 'adult stem cells' OR 'somatic stem cell' OR 'mother cells' OR 'colony forming unit'/exp OR 'cfu' OR 'colony form unit' OR 'colony forming ability' OR 'colony forming capacity' OR 'colony forming unit' OR 'embryonic stem cell'/exp OR 'es cell' OR 'embryonic stem cell' OR 'embryonic stem cells' OR 'stem cell' OR 'adipose derived stem cell'/exp OR 'endothelial progenitor cell'/exp OR 'stem cell'/exp OR 'epidermal stem cell'/exp OR 'epidermal stem cell' OR 'hematopoietic stem cell'/exp OR 'bone marrow stem cell' OR 'haematopoietic precursor cell' OR 'haematopoietic progenitor cell' OR 'haematopoietic stem cell' OR 'haematopoietic stem cells' OR 'hematocytopoietic stem cell' OR 'hematopoietic precursor cell' OR 'hematopoietic progenitor cell' OR 'hematopoietic stem cell' OR 'hematopoietic stem cells' OR 'hemocytopoietic stem cell' OR 'hemopoietic stem cell' OR 'mesenchymal stem cell'/exp OR 'mesenchymal progenitor cell' OR 'mesenchymal stem cell' OR 'mesenchymal stem cells' OR 'stem cell, mesenchymal' OR 'multipotent stem cell'/exp OR 'multipotent cell' OR 'multipotent precursor cell' OR 'multipotent progenitor cell' OR 'multipotent stem cell' OR 'multipotent stem cells' OR 'peripheral blood stem cell'/exp OR 'peripheral blood precursor cell' OR 'peripheral blood progenitor cell' OR 'peripheral blood stem cell' OR 'peripheral blood stem cells' OR 'stem cell, peripheral blood' OR 'whartons jelly mesenchymal stem cells':ti,ab OR 'whartons jelly cells':ti,ab OR 'human umbilical cord mesenchymal stem cells':ti,ab OR 'skin derived stem cells':ti,ab OR MSC:ti,ab OR ADCS:ti,ab) AND ('wound healing'/exp OR 'granulation,	12/18/2023	10431

	wound' OR 'healing, wound' OR 'repair, wound' OR 'wound granulation' OR 'wound healing' OR 'wound regeneration' OR 'wound repair' OR 'platelet derived wound healing' OR 'chronic wound'/exp OR 'chronic wound' OR 'chronic wounds' OR 'wounds, chronic' OR 'diabetic wound'/exp OR 'diabetic dermal wound' OR 'diabetic skin wound' OR 'diabetic wound') NOT 'animal experiment'		
Web of Science	"Wound healing" OR "platelet derived wound healing" OR "wound regeneration" (All Fields) and "Stem Cells" OR "Adult Stem Cells" OR "Embryonic Stem Cells" OR "Stem Cell Transplantation" OR "Adipose Tissue/cytology" OR "Mesenchymal Stem Cells" OR "Pluripotent Stem Cells" OR "Mesenchymal Stem Cell Transplantation" OR "stem cell" OR "Progenitor Cells" OR "mother cells" OR "Colony forming units" OR "adult stem cells" OR "Adipose tissue derived stem cells" OR "bone marrow derived mesenchymal stem cells" OR "inducible pluripotent stem cells" OR "endothelial progenitor stem cells" OR "epidermal stem cells" OR "peripheral blood derived mesenchymal stem cells" OR "human umbilical cord mesenchymal stem cells" OR "skin derived stem cells" OR "Wharton's jelly mesenchymal stem cells" OR "Wharton's Jelly Cells" OR "Mesenchymal stromal cells" OR MSC OR ADCS (All Fields) and human OR humans (All Fields)	12/18/2023	4785
Cochrane Library Clinical Trials	#1 MeSH descriptor: [Wound Healing] explode all trees<br>#2 "wound healing" OR "Platelet derived wound healing" OR "wound regeneration"<br>#3 MeSH descriptor: [Stem Cells] explode all trees<br>#4 MeSH descriptor: [Adult Stem Cells] explode all trees<br>#5 MeSH descriptor: [Embryonic Stem Cells] explode all trees<br>#6 MeSH descriptor: [Stem Cell Transplantation] explode all trees<br>#7 MeSH descriptor: [Mesenchymal Stem Cells] explode all trees<br>#8 MeSH descriptor: [Pluripotent Stem Cells] explode all trees<br>#9 MeSH descriptor: [Mesenchymal Stem Cell Transplantation] explode all trees<br>#10 "stem cell" OR "Progenitor Cells" OR "mother cells" OR "Colony forming units" OR "adult stem cells" OR "Adipose tissue derived stem cells" OR "bone marrow derived mesenchymal stem cells" OR "inducible pluripotent stem cells" OR "endothelial progenitor stem cells" OR "epidermal stem cells" OR "peripheral blood derived mesenchymal stem cells" OR "human umbilical cord mesenchymal stem cells" OR "skin derived stem cells" OR "Wharton's jelly mesenchymal stem cells" OR "Wharton's Jelly Cells" OR "Mesenchymal stromal cells" OR MSC OR ADCS<br>#11 human or humans<br>#12 #1 OR #2<br>#13 #3 OR #4 Or #5 Or #6 OR #7 OR #8 OR #9 OR #10<br>#14 #12 AND #11 AND #13	12/18/2023	202
Google Scholar	("Wound healing" OR "platelet derived wound healing" OR "wound regeneration") AND ("Stem Cells" OR "Adult Stem Cells" OR "Embryonic Stem Cells" OR "Stem Cell Transplantation" OR "Adipose Tissue/cytology" OR "Mesenchymal Stem Cells" OR "Pluripotent Stem Cells" OR "Mesenchymal Stem Cell Transplantation" OR	12/18/2023	First 300 when

	"stem cell" OR "Progenitor Cells" OR "mother cells" OR "Colony forming units" OR "adult stem cells" OR "Adipose tissue derived stem cells" OR "bone marrow derived mesenchymal stem cells" OR "inducible pluripotent stem cells" OR "endothelial progenitor stem cells" OR "epidermal stem cells" OR "peripheral blood derived mesenchymal stem cells" OR "human umbilical cord mesenchymal stem cells" OR "skin derived stem cells" OR "Wharton's jelly mesenchymal stem cells" OR "Wharton's Jelly Cells" OR "Mesenchymal stromal cells" OR MSC OR ADCS) AND (human or humans)		sorted by relevancy
